# Supplementary material for: Long-range allosteric regulation of the human 26S proteasome by 20S proteasome-targeting cancer drugs
Source: Nat Commun. 2017 May 25;8:15578. doi: 10.1038/ncomms15578 (PMC5458511; doi:10.1038/ncomms15578)
Supplement: Supplementary Information — Supplementary Figures, Supplementary Tables, Supplementary Note [file ncomms15578-s1.pdf]

Supplementary Information: Long range allosteric regulation of the human 26S proteasome by 20S proteasome-targeting cancer drugs

David Haselbach*, Jil Schrader*, Felix Lambrecht, Fabian Henneberg, Ashwin Chari and Holger Stark

Supplementary Note

In the study presented in the main text, we show energy landscapes from cryo-EM data. This supplemental text aims to outline the underlying concept and to describe the method we developed to generate those landscapes.

If not stated differently, particles with and without Oprozomib bound to the proteasome were treated as separate subsets. All particles of one subset were refined against the initial 26 S proteasome map using RELION to gain alignment parameters. This reduces the subsequent calculations in the classification step. The resulting aligned images were randomly split in subsets of 100,000 particles and a RELION 3D-classification[1], yielding 40 classes per subset, was performed, without aligning the particles again. Each resulting 3D class was further refined with the assigned particles using RELION.

To further analyze the motions between the in total 346 classes we used principle component analysis (PCA). The result of a PCA are eigenvectors e_j , that describe the largest covariance within the dataset. In aligned 3D volumes of the same molecule, the largest covariance are primarily movements within the molecule[2]. The eigenvectors can be used as conformational coordinates.[3] Before applying PCA, the refined 3D-classes were aligned in UCSF Chimera against a model of the most rigid part of the 26S complex, the 20 S proteasome subcomplex. This is necessary to avoid calculating eigenvectors which describe shifts and rotations of the 3D-classes among themselves. 3D classes from datasets with and without Oprozomib were combined and normalized, and subsequently eigenvectors were calculated using PCA. Hence, the eigenvectors describe the movements found in both datasets and allow us to compare the results in the end.

One can then describe the conformers X_i on a coordinate e_n (the eigenvector describing movement n) by:

$$X_i = a_{n,i} e_n + \bar{X} \qquad (1)$$

where $\bar{X}$ is the average volume, e_n is a specific eigenvector and $a_{n,i}$ is a linear factor. In other words, e_n is a conformational coordinate and $a_{n,i}$ places the conformer X_i at its specific place on this conformational coordinate. The addition of the mean volume results from the definition of PCA. Eigenvectors and hence their corresponding trajectories can be sorted according to their contribution to the overall mobility.

However, the movements described by the conformational coordinates might be composed out of several primitive motions of the molecule. Therefore, one cannot assign a single measure (e.g. rotation angle, translation movement) to all of those movements. The following movements where found and are listed descending in the value of the covariance:

Eigenvector	Description
1	Rotation of Lid and translation of ATPase
2	Resolution differences
3	conformational stabilization of Rpn9
4	conformational stabilization of Rpn5,10 and 12, movement of Rpn1
5	Motion of Rpn1, conformational stabilization of Rpn 9,10 , small breathing of lid
6	Translation of Rpn2, 3, 7, 12
7	Stretching of lid, movement of central helix bundle(Rpn3, 6, 8, 11), movement of ATPase
8	Rise of ATPase (including Rpn1), sinking of Rpn2, 8
9	Rotation of Rpn1
10	Small movements

To understand the motion described by the eigenvectors, video trajectories where interpolated using equation (1) and subsequent morphing in UCSF Chimera (see Supplementary video 1). Then, for each 3D-class the linear factors with respect to each eigenvector were determined.

By placing the different conformers on the reaction coordinates energetic conclusions can be drawn. Knowing the number of particles assigned to each class, we can calculate their free energy differences $\Delta\Delta G$ by the Boltzmann Factor:

$$\Delta\Delta G = k_B T \ln\left(\frac{p_i}{p_0}\right) \quad (2)$$

, where T is the absolute temperature, p_i is the number of particles in state i and p_0 is the number of particles in the most populated state [4]. The number of particles belonging to each 3D

was counted from the respective data output files from RELION. Free energy differences were calculated using equation (2) as multiples of $k_B T$. From equation (2) it becomes obvious that regions with high energies have a lower number of single particles belonging to them. To visualize the energy landscape with and without Oprozomib, combinations of two eigenvectors e_n, e_m were used as the axis of a new three-dimensional coordinate system. The 3D classes were placed as data points in these landscapes with the dimensions being the respective linear factors $a_{n,i}$and $a_{m,i}$, and the difference in free energy $\Delta\Delta G$. By Interpolating between those discrete states, we could in the end describe energy landscapes in which the molecule moves.

To account for false-positives, i.e. 3Ds which are classified in two separate classes but do only differ slightly, we applied a final binning of close data points. Therefore, all data points within a given distance were averaged. This distance was set to the half-width of the peak around one linear factor.

These landscapes have important limitations. In contrast to e.g. Molecular Dynamics simulations, the 3D volumes yielded from single particle analysis sample the conformational space discrete and sparse. Hence, large areas in the landscapes – especially those of high energy - are highly interpolated. This also represents in the fact that the very low sampled areas close to unfolding and complex decomposition are not accessible to the method.

However, the method offers an opportunity to quantify the results of 3D classifications and, as in our specific case, allows to learn about the influence of small molecule binding on the functional conformational landscape of a macromolecular machine.

Supplementary References

1. Scheres, S. H. W. *Processing of Structurally Heterogeneous Cryo-EM Data in RELION*. *The Resolution Revolution: Recent Advances In cryoEM* (Elsevier Inc., 2016). doi:10.1016/bs.mie.2016.04.012

2. Penczek, P. a, Kimmel, M. & Spahn, C. M. T. Identifying conformational states of macromolecules by eigen-analysis of resampled cryo-EM images. *Structure* **19,** 1582–90 (2011).

3. Zhuravlev, P. I. & Papoian, G. A. Protein functional landscapes, dynamics, allostery: a tortuous path towards a universal theoretical framework. *Q. Rev. Biophys.* **43,** 295–332 (2010).

4. Beveridge, D. L. & DiCapua, F. M. Free energy via molecular simulation: applications to chemical and biomolecular systems. *Annu. Rev. Biophys. Biophys. Chem.* **18,** 431–92 (1989).

Supplementary Figures and tables

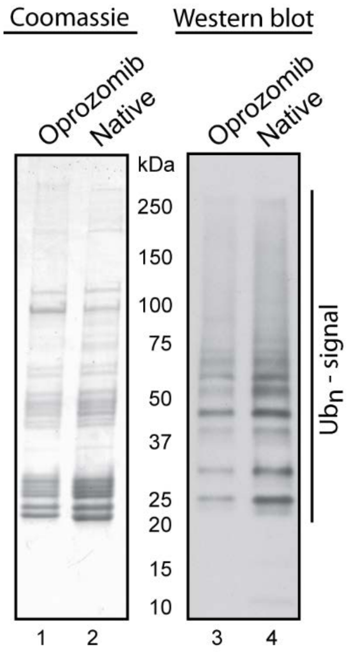

Supplementary Figure 1: Western Blot against ubiquitin

We measured the level of polyubiquitinated substrate bound to the purified 26S proteasome by SDS PAGE and Western Blot analysis using an antibody directed against ubiquitin. Similar ubiquitin levels were detected for the inhibited (Oprozomib) and the non-inhibited (native) proteasome.

Cryo Statistics

**Micrographs recorded:**	**18.991**
**Picked particles:**	**2.521.909**
after CTF & classification cleaning:	924.068

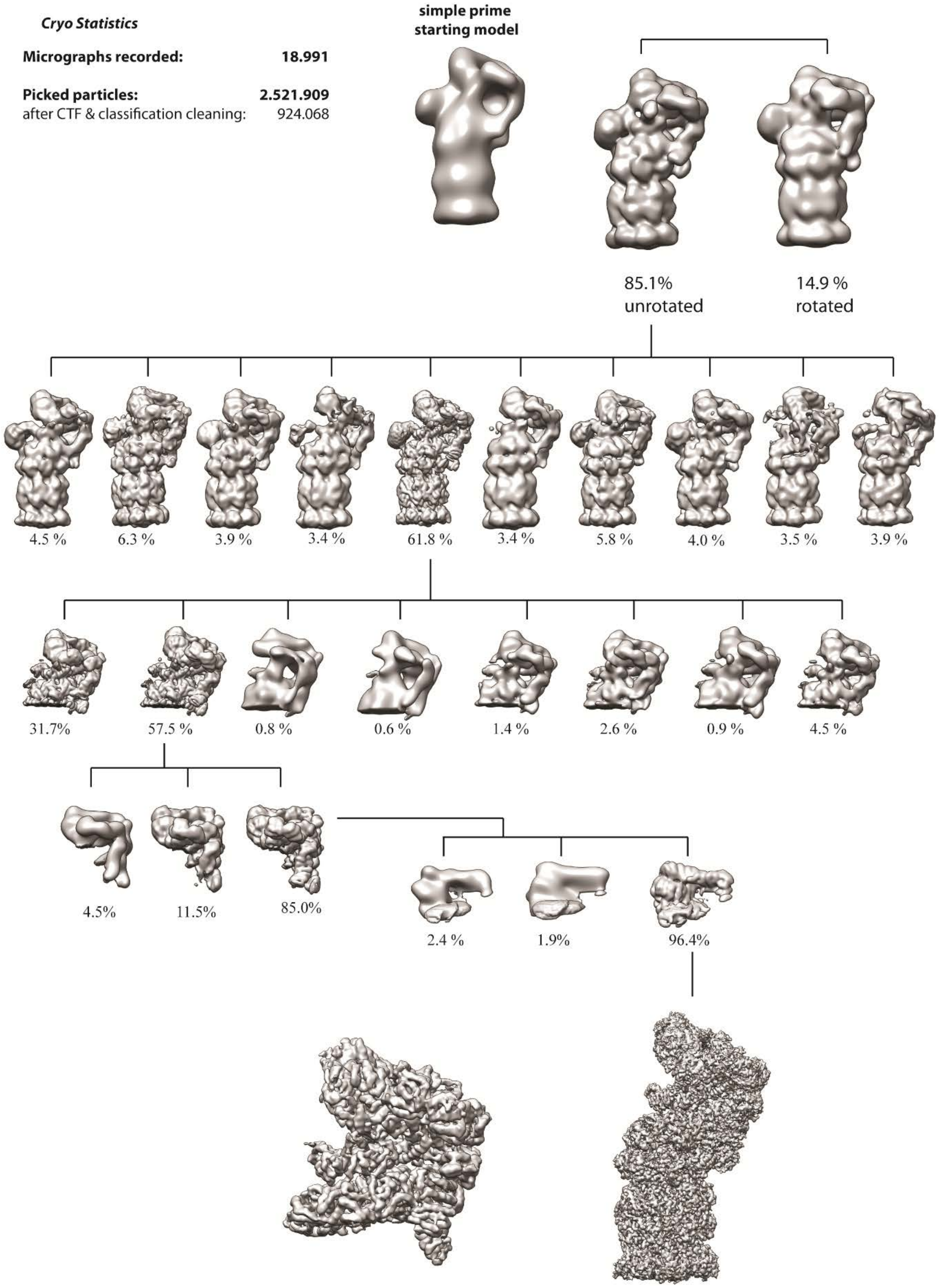

Supplementary Figure 2: Computational Particle Image Sorting scheme

The scheme shows the various computational image sorting steps required for the determination of the 3.8 Å resolution structure. First an initial 3D model was calculated using the software Simple PRIME. In an initial 3D classification particles were sorted with respect to the two main rotational conformations of the RP. Particles contributing to the non-rotated state were further classified. The highest populated class has been classified by focused classification using a mask for the 19S subcomplex. The best classes were chosen and two consecutive rounds of focused classification have been performed using a mask for the lid subcomplex and finally a mask for Rpn2. The remaining particles were refined in RELION to 3.8 Å. Furthermore, the signal contributing to the 20S density was computationally removed from the raw images in RELION and the remaining 19S subcomplex was refined to 4.1 Å

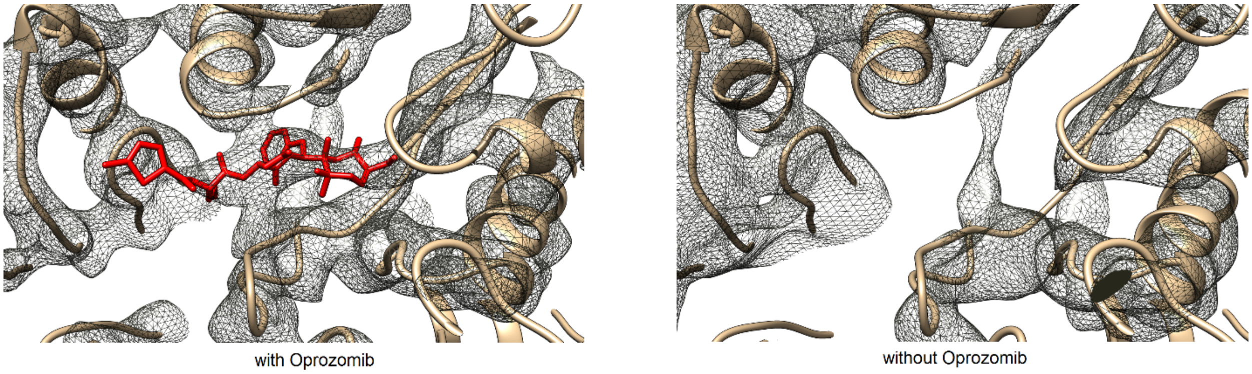

Supplementary Figure 3: Oprozomib binding site in a low-pass filtered density at 4.8 Å resolution

Close-up view of the Oprozomib binding site in the β5 subunit of the CP. This is essentially the same Figures like Fig. 1f and 1g. In contrast to Fig. 1f both maps are shown at the same level of resolution (4.8 Å) to allow direct comparison and to avoid potential resolution effects.

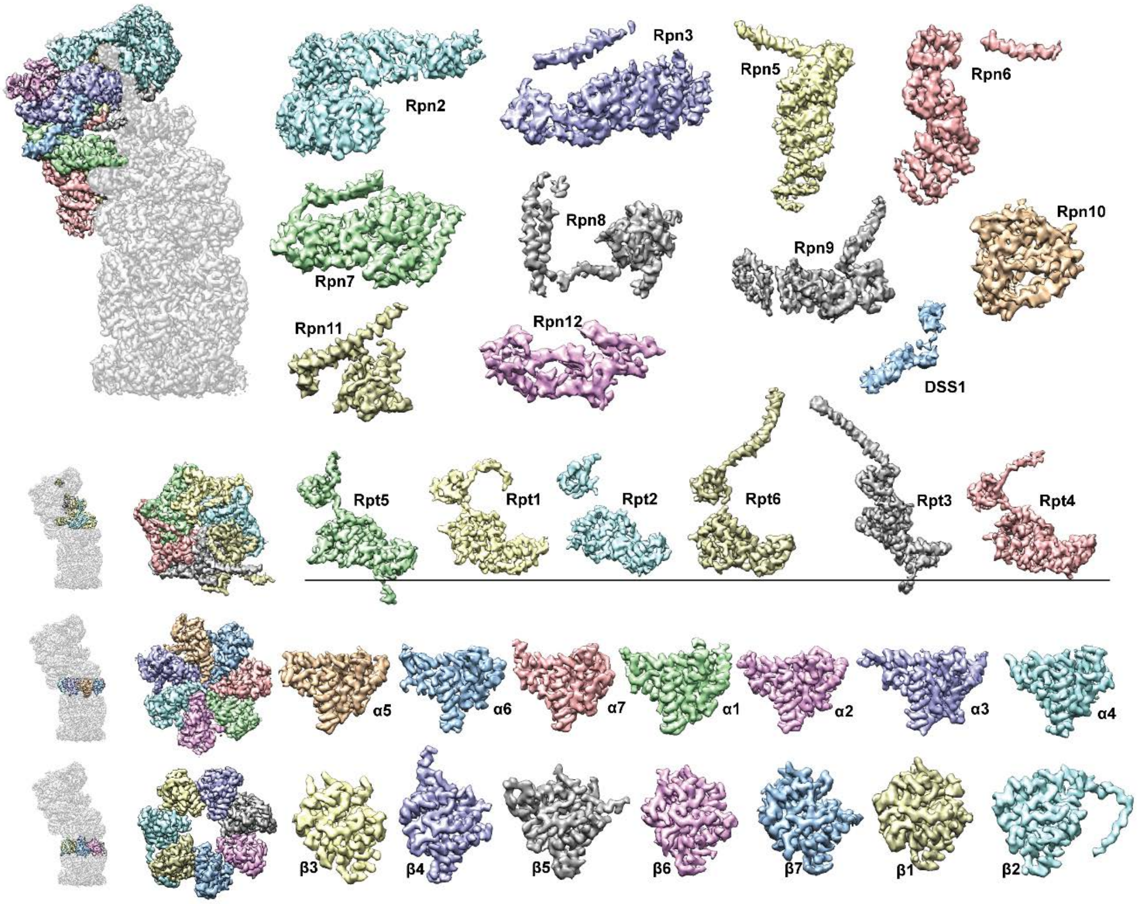

Supplementary Figure 4: Representation of densities, corresponding to the modeled subunits

Segmentation of all 26S proteasome components (Supplementary Table 1) sorted by lid proteins (upper rows), ATPase, α and β subunits (lower three rows). The horizontal line below the ATPase (Rpt) subunits indicates the surface plane of the CP.

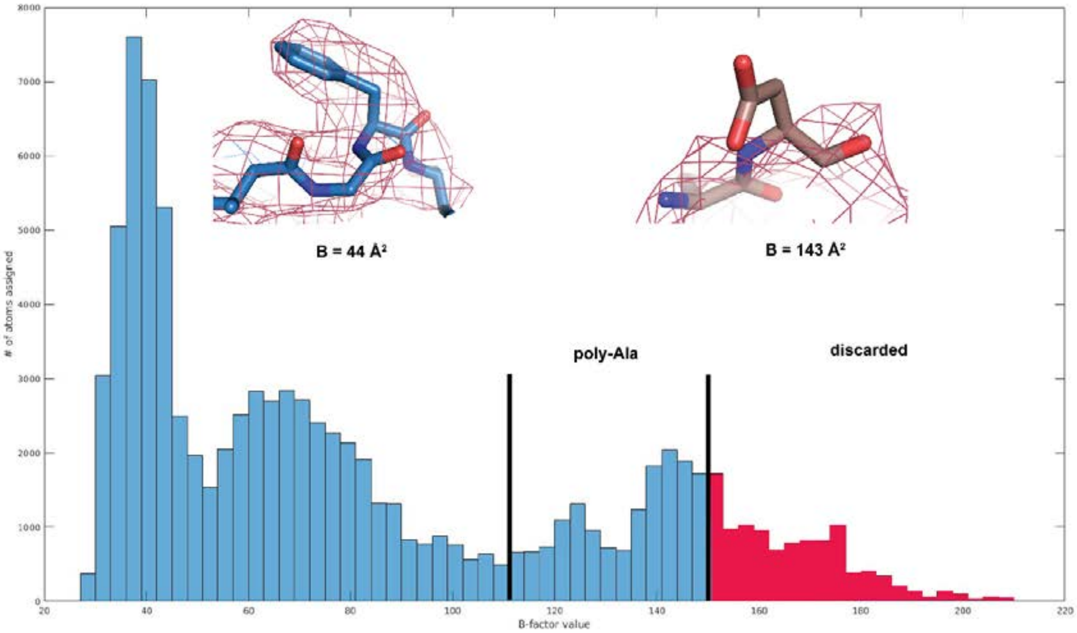

Supplementary Figure 5: B-Factor Distribution

We obtained model B-factors by an atomic displacement factor refinement in Phenix and defined three quality levels, which were used to decide about the validity of building side chains into various regions of the final 3.8 Å resolution structure. Side chains are in general clearly visible in areas of the 3D density with B factors <110 $Å^2$. For segments with B-Factors in the range of 110 $Å^2$ < B < 150 $Å^2$ side chains are no longer clearly visible but there is sufficient density for the protein backbone. Regions with B factors higher than 150 $Å^2$ are not suitable to build reliable models at all. We therefore included side chains in the deposited model only for those parts of the 3D map where B factors <110 $Å^2$ were obtained. In our model, the protein backbone is included for B factors 110 $Å^2$ < B < 150 $Å^2$ and no model was built for B factors >150 $Å^2$.

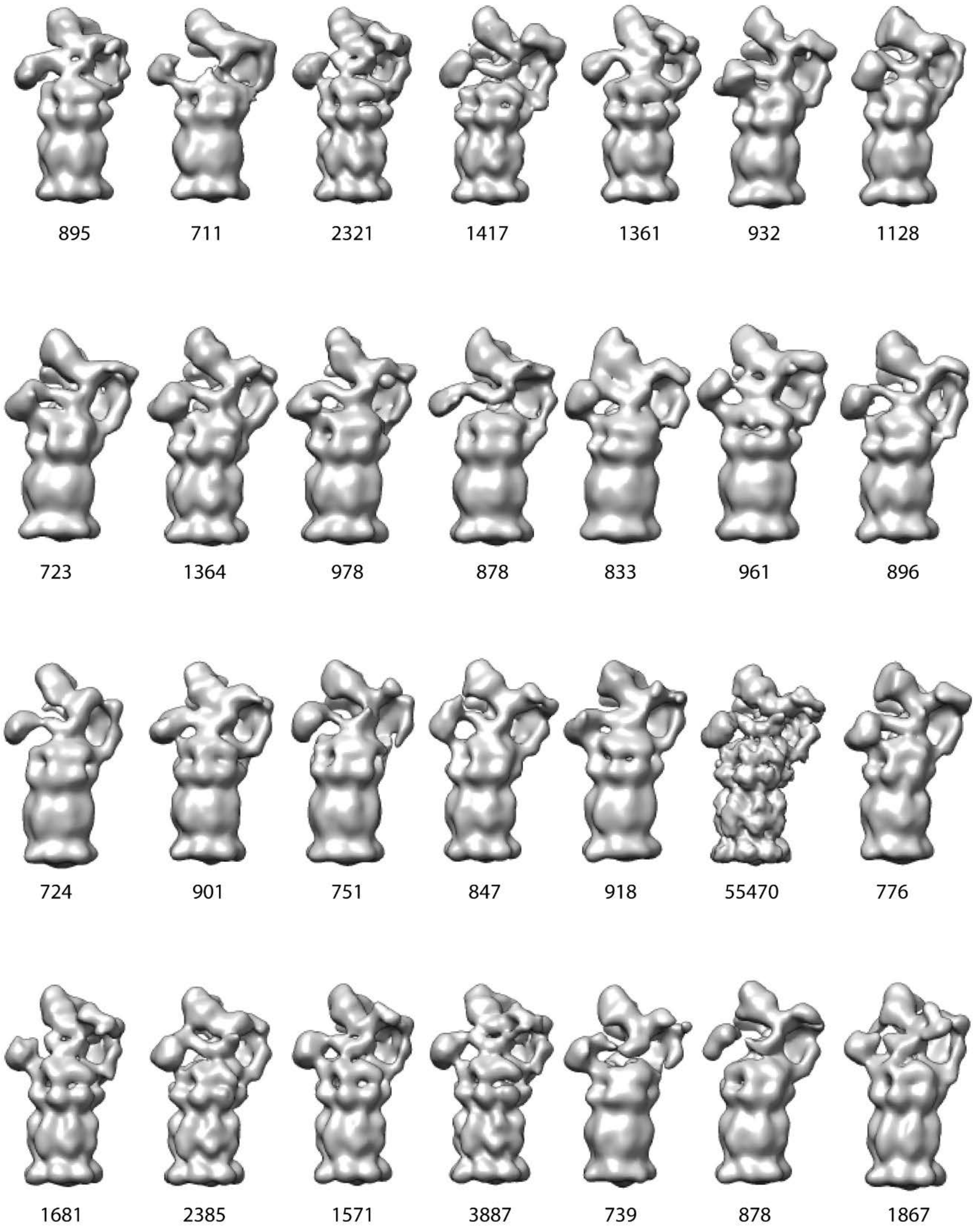
895
711
2321
1417
1361
932
1128
723
1364
978
878
833
961
896
724
901
751
847
918
55470
776
1681
2385
1571
3887
739
878
1867

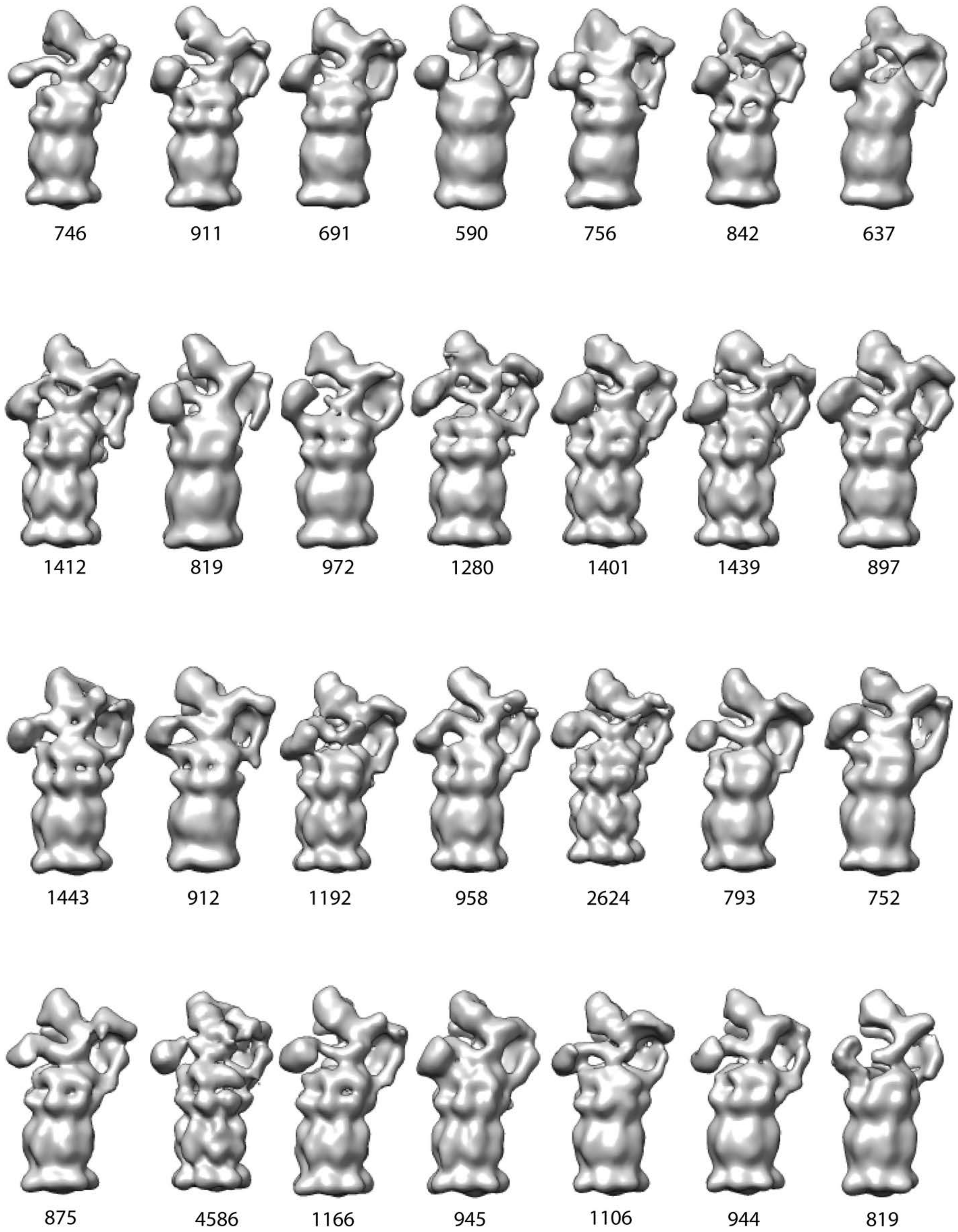
746
911
691
590
756
842
637
1412
819
972
1280
1401
1439
897
1443
912
1192
958
2624
793
752
875
4586
1166
945
1106
944
819

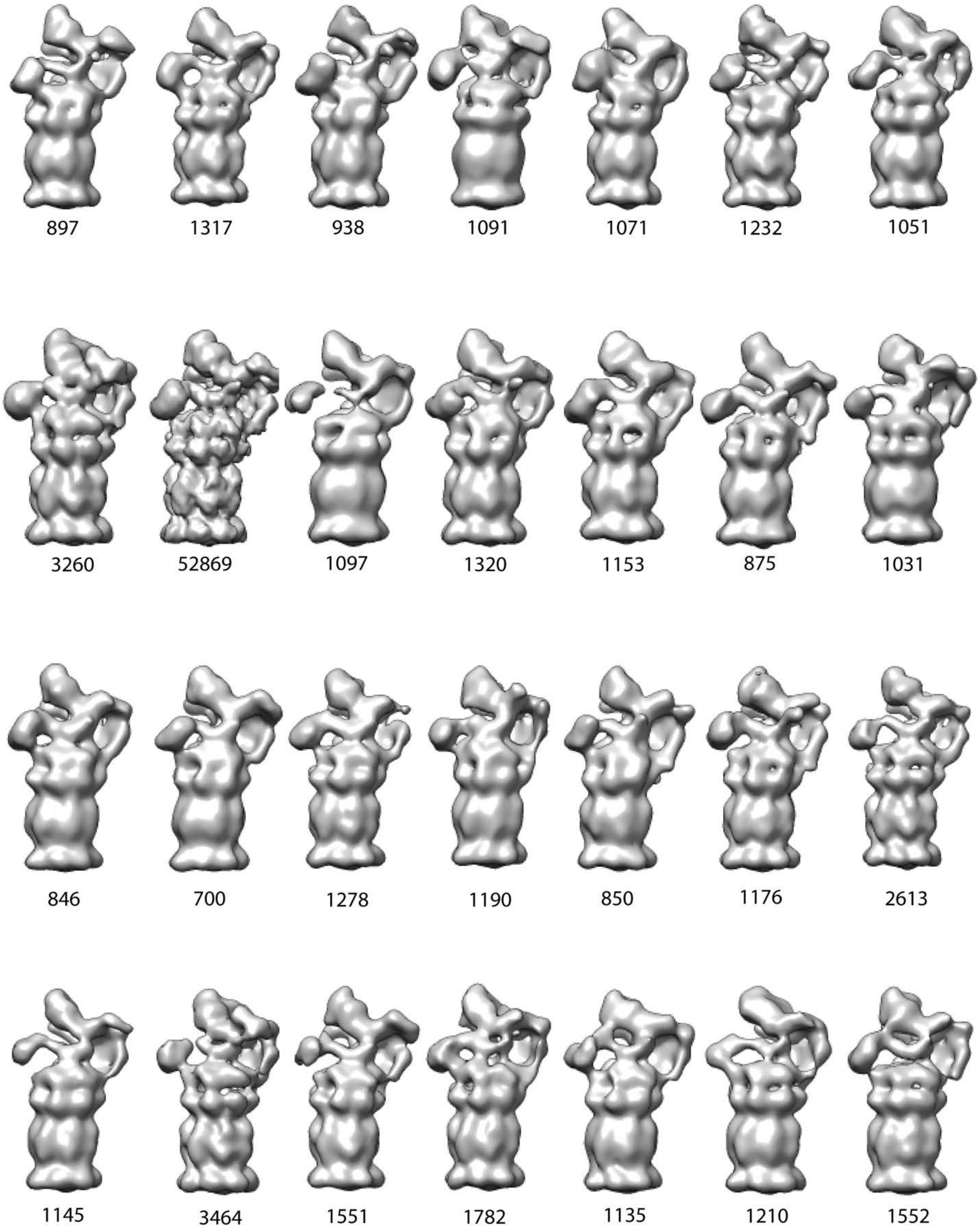
897
1317
938
1091
1071
1232
1051
3260
52869
1097
1320
1153
875
1031
846
700
1278
1190
850
1176
2613
1145
3464
1551
1782
1135
1210
1552

165

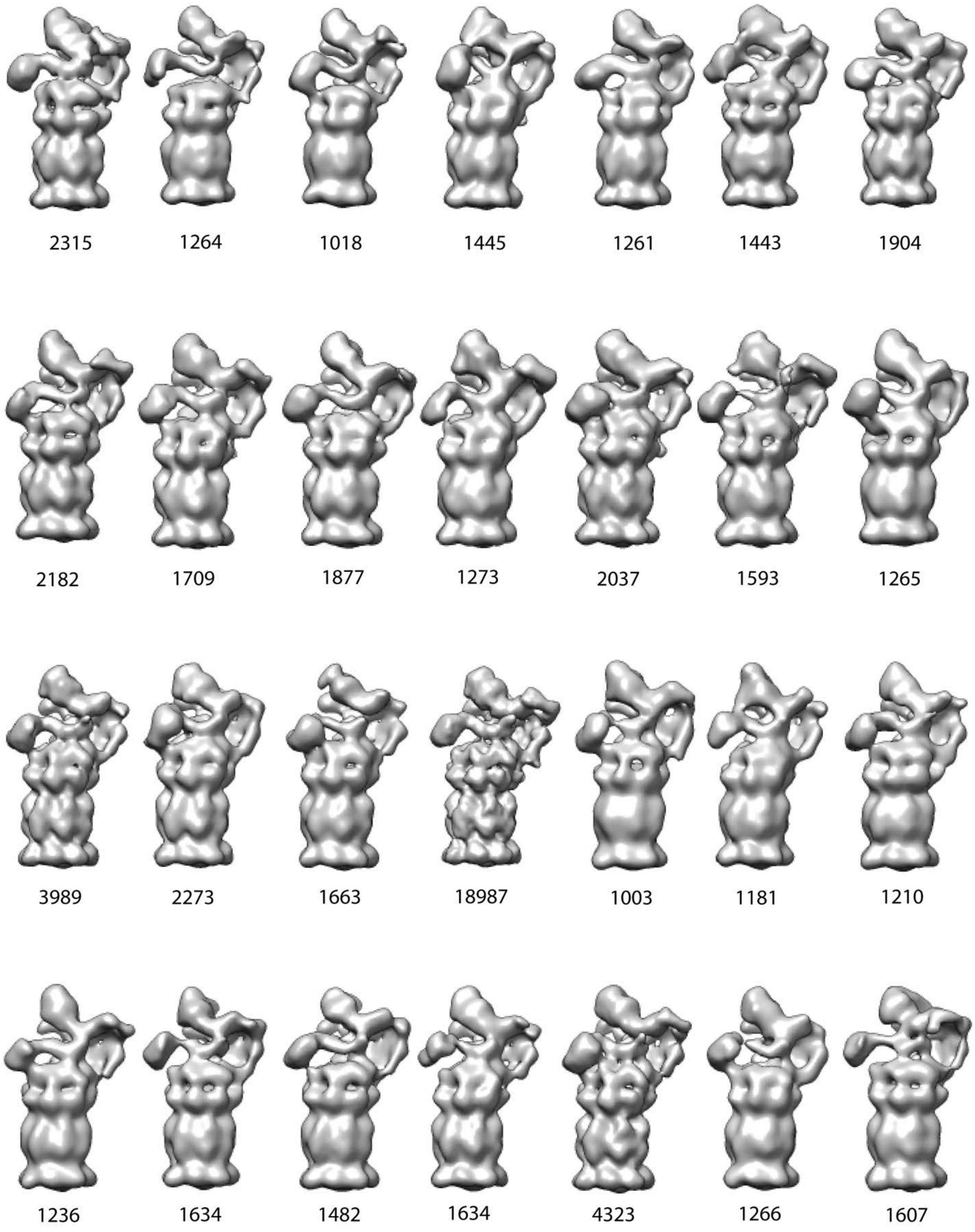
2315
1264
1018
1445
1261
1443
1904
2182
1709
1877
1273
2037
1593
1265
3989
2273
1663
18987
1003
1181
1210
1236
1634
1482
1634
4323
1266
1607

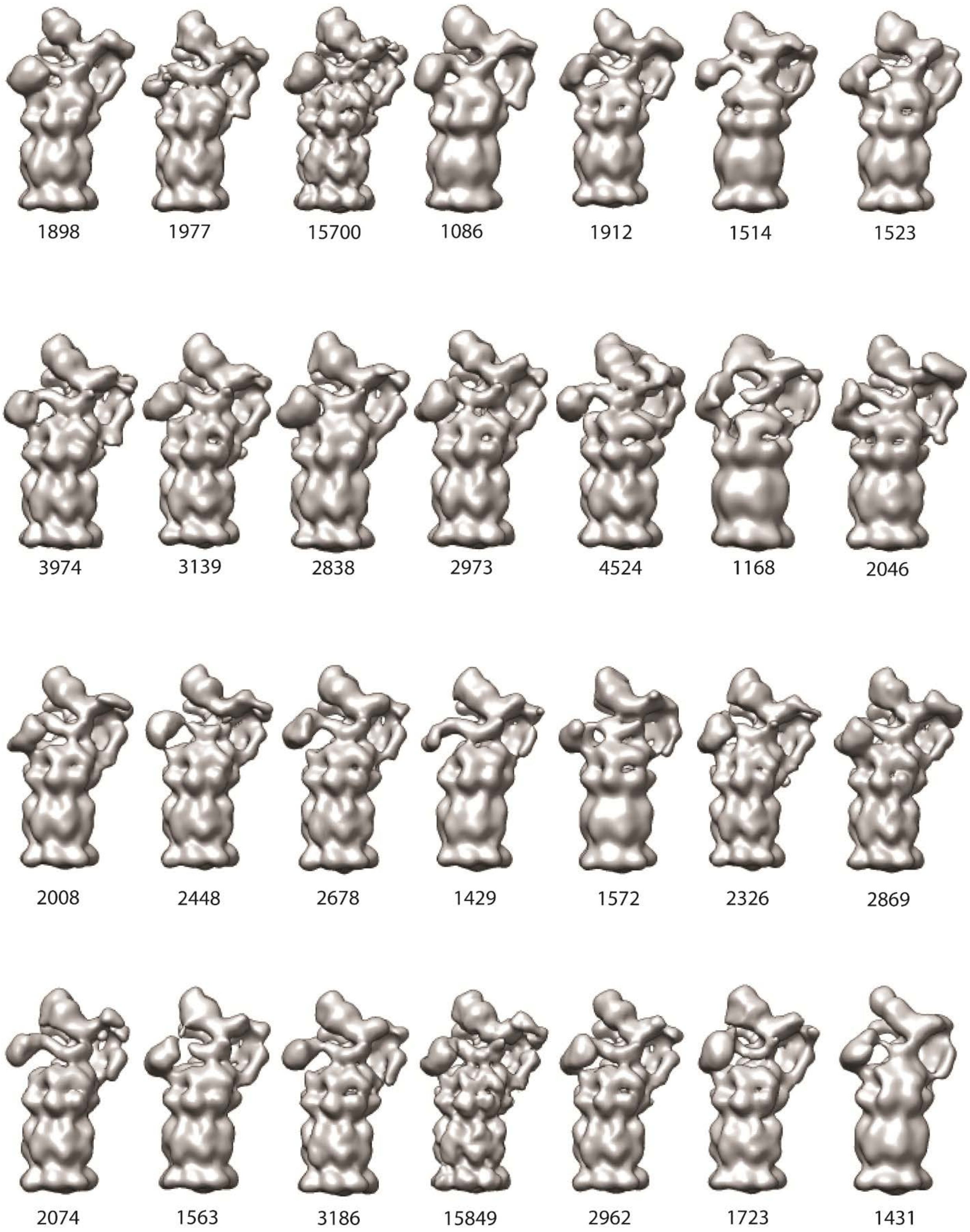

1898
1977
15700
1086
1912
1514
1523
3974
3139
2838
2973
4524
1168
2046
2008
2448
2678
1429
1572
2326
2869
2074
1563
3186
15849
2962
1723
1431

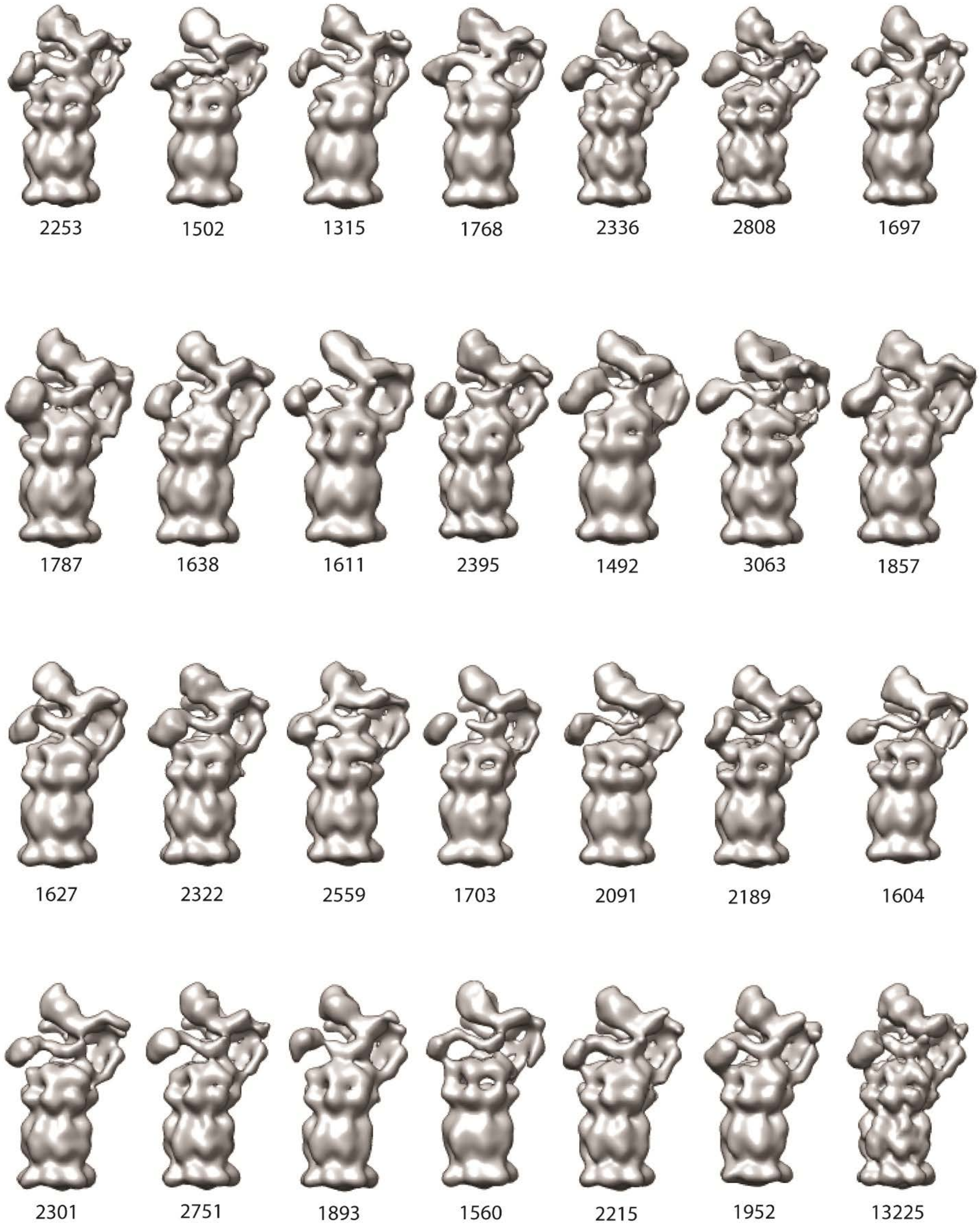

2253
1502
1315
1768
2336
2808
1697
1787
1638
1611
2395
1492
3063
1857
1627
2322
2559
1703
2091
2189
1604
2301
2751
1893
1560
2215
1952
13225

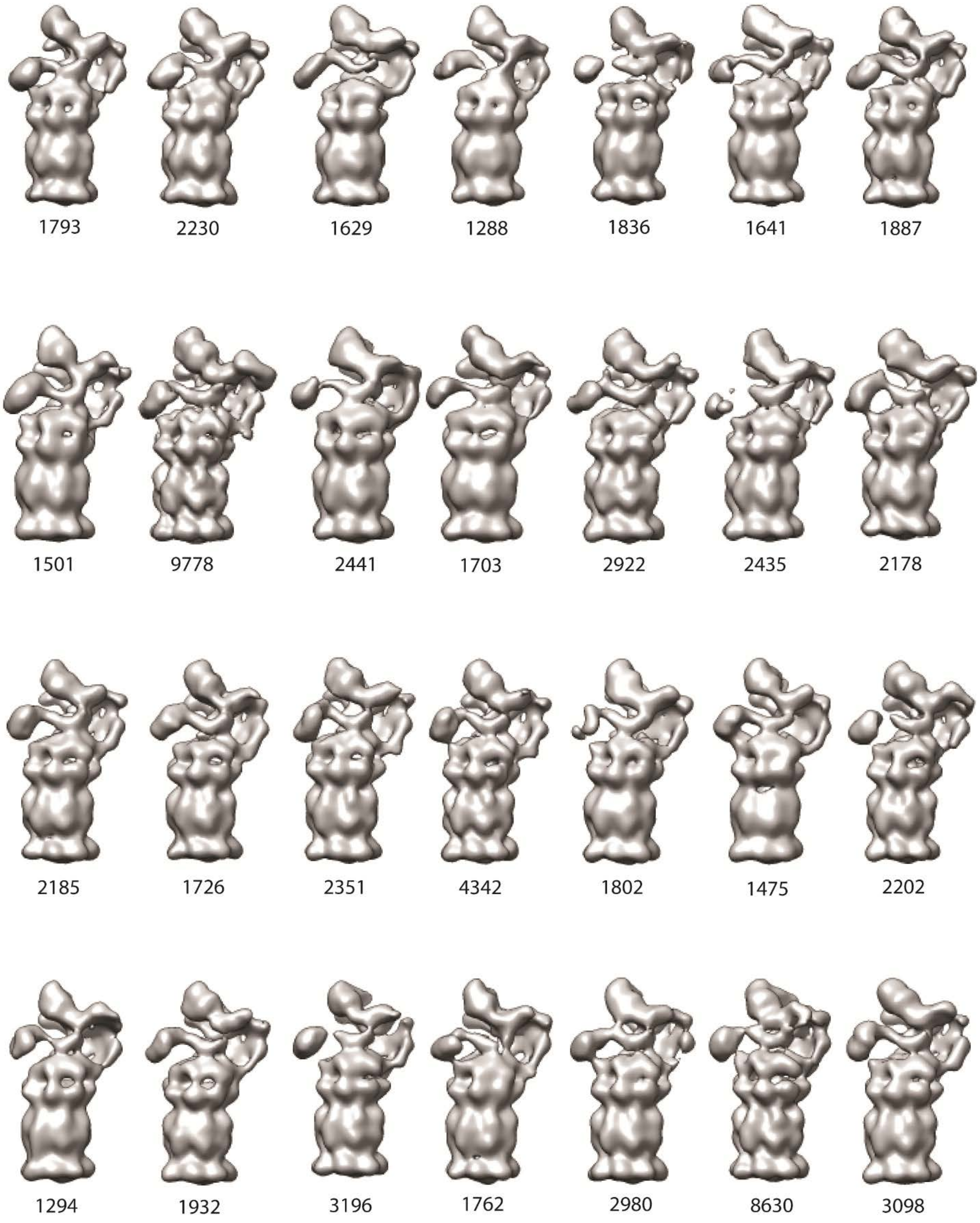
1793
2230
1629
1288
1836
1641
1887
1501
9778
2441
1703
2922
2435
2178
2185
1726
2351
4342
1802
1475
2202
1294
1932
3196
1762
2980
8630
3098

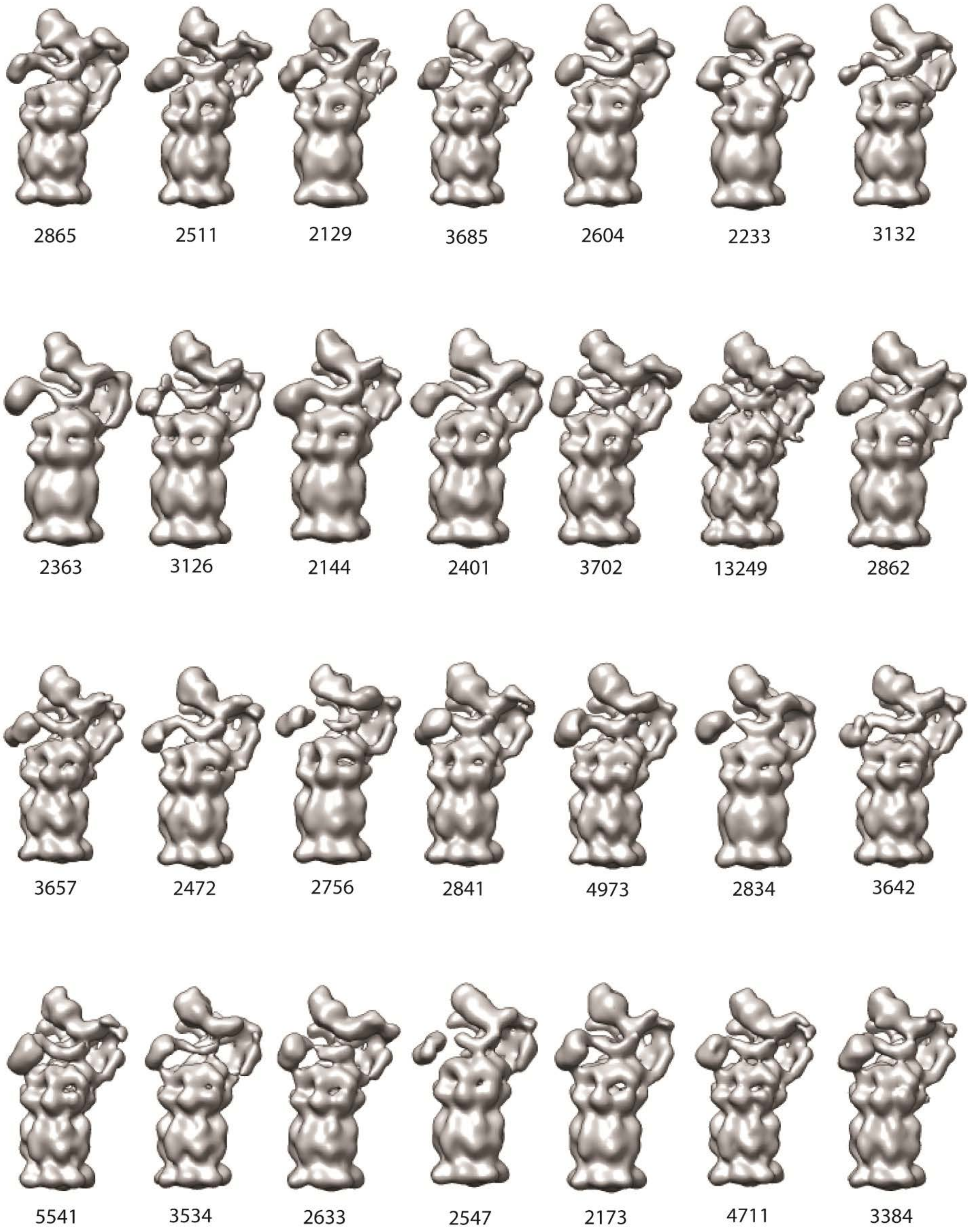
2865
2511
2129
3685
2604
2233
3132
2363
3126
2144
2401
3702
13249
2862
3657
2472
2756
2841
4973
2834
3642
5541
3534
2633
2547
2173
4711
3384

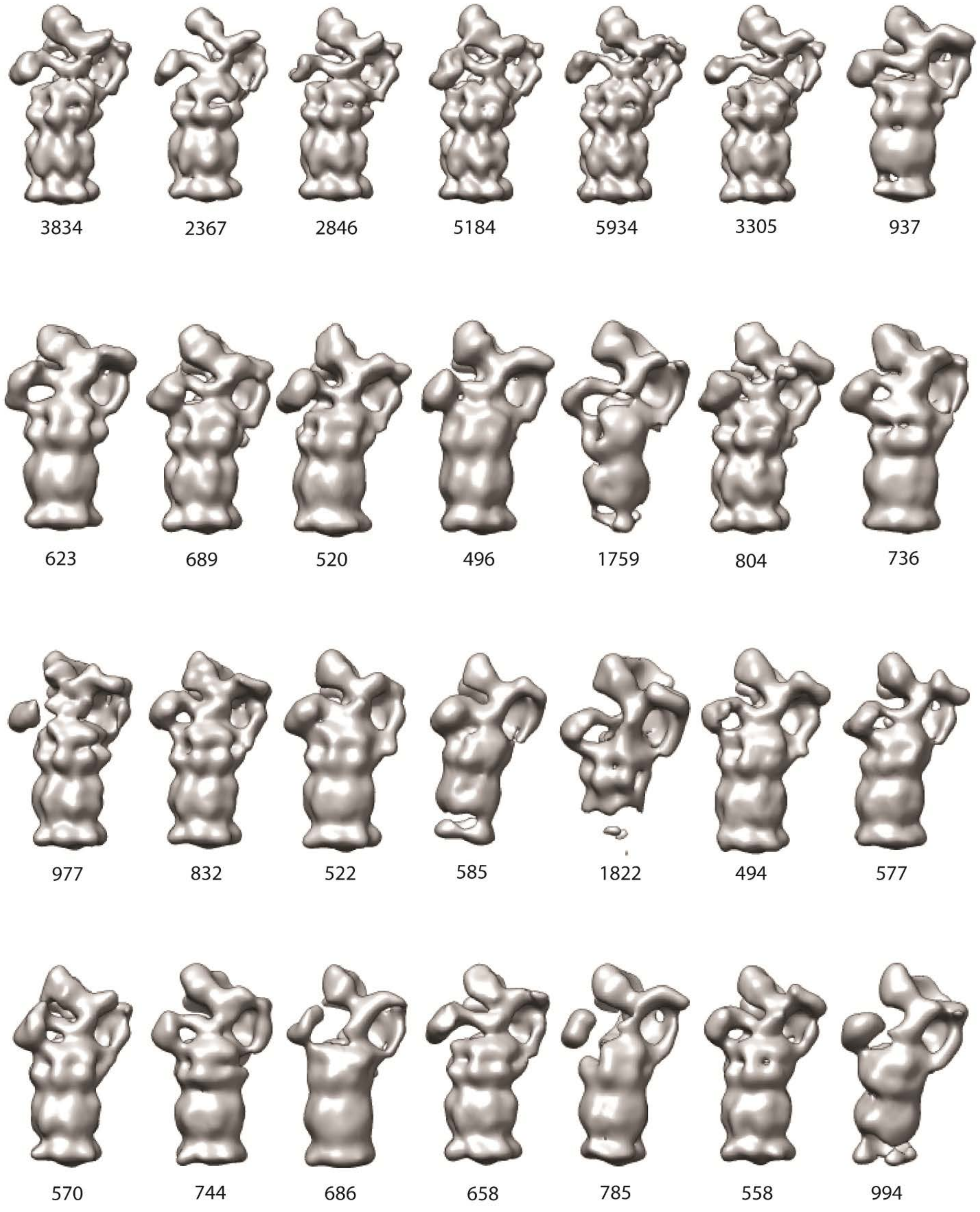
3834
2367
2846
5184
5934
3305
937
623
689
520
496
1759
804
736
977
832
522
585
1822
494
577
570
744
686
658
785
558
994

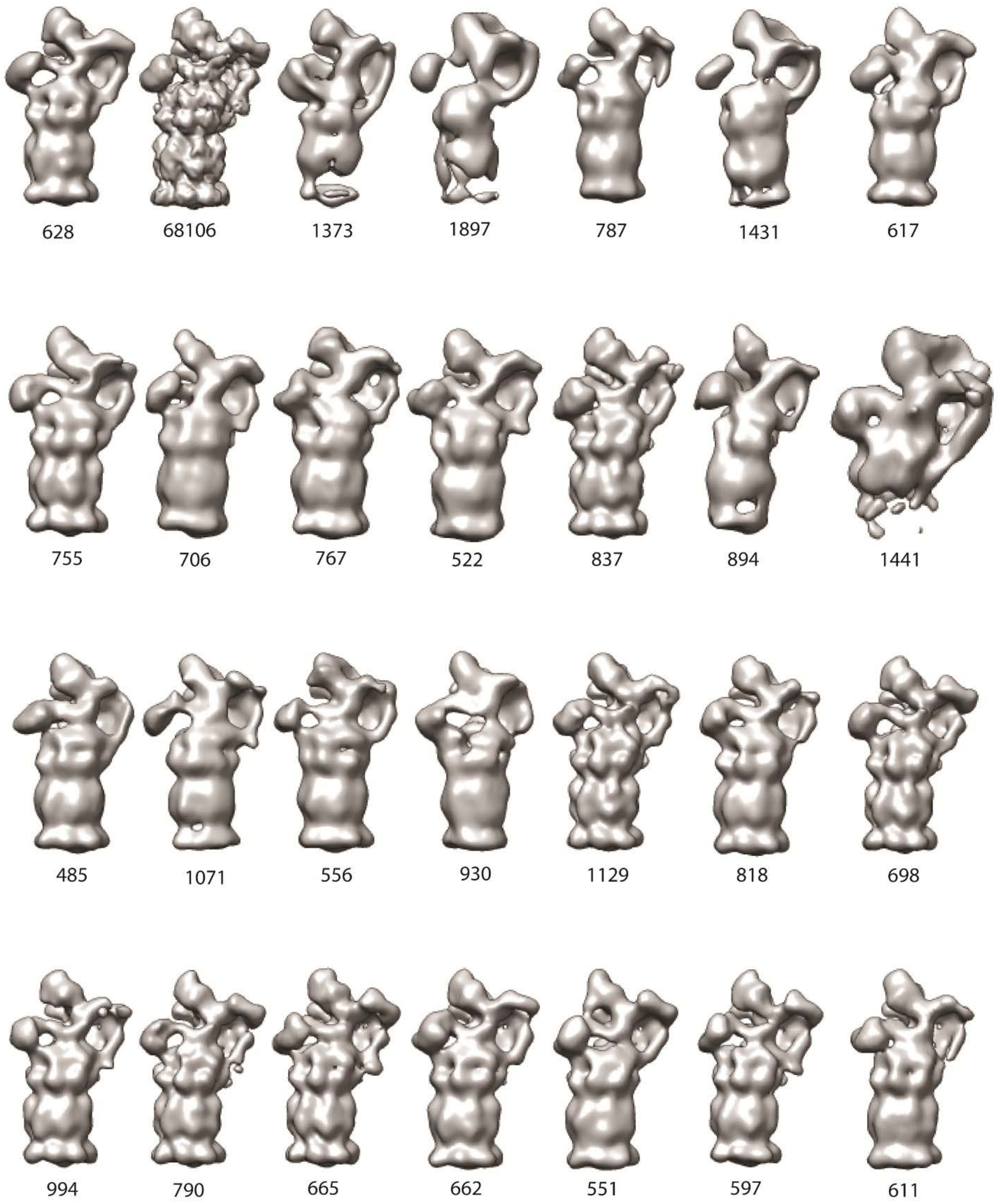
628
68106
1373
1897
787
1431
617
755
706
767
522
837
894
1441
485
1071
556
930
1129
818
698
994
790
665
662
551
597
611

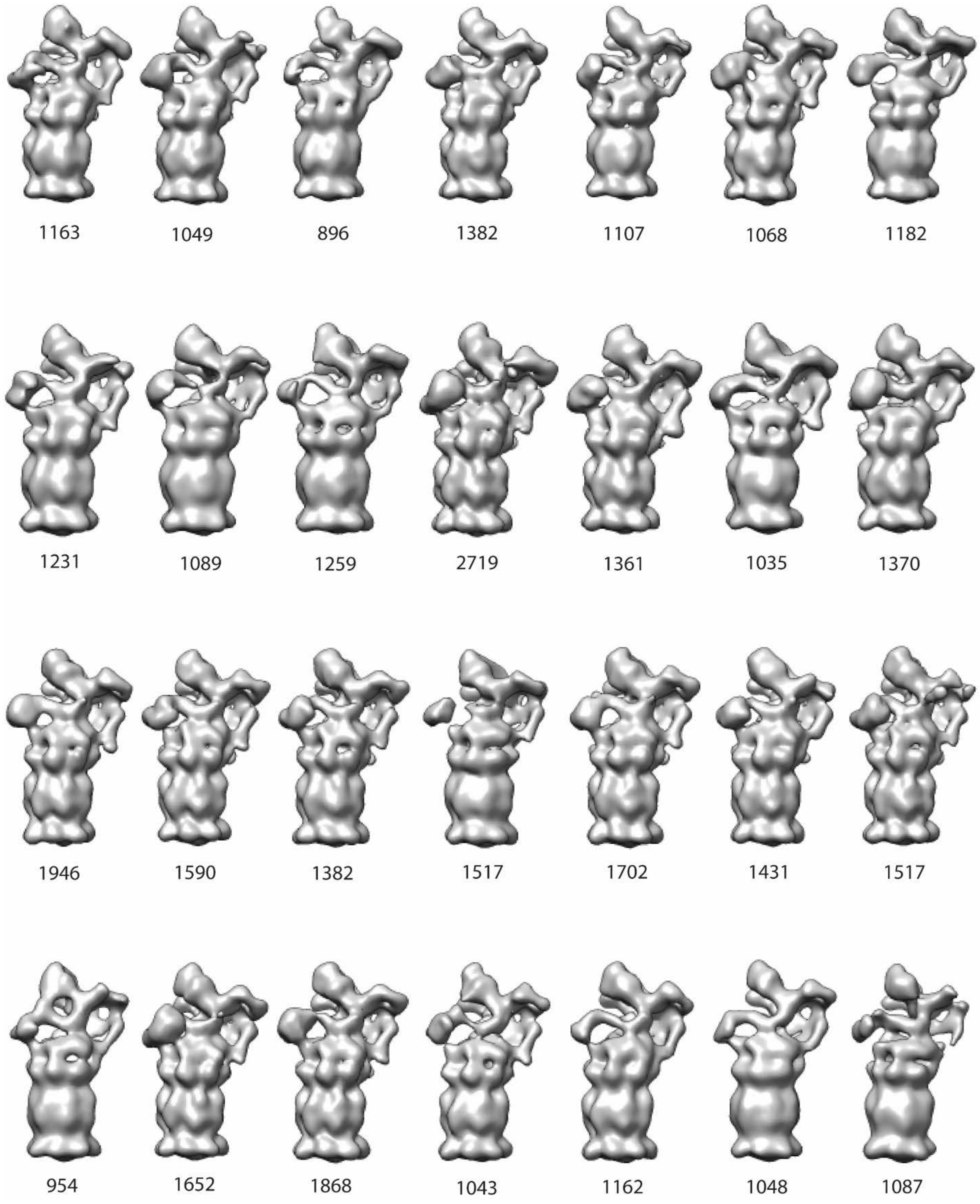
1163
1049
896
1382
1107
1068
1182
1231
1089
1259
2719
1361
1035
1370
1946
1590
1382
1517
1702
1431
1517
954
1652
1868
1043
1162
1048
1087

175

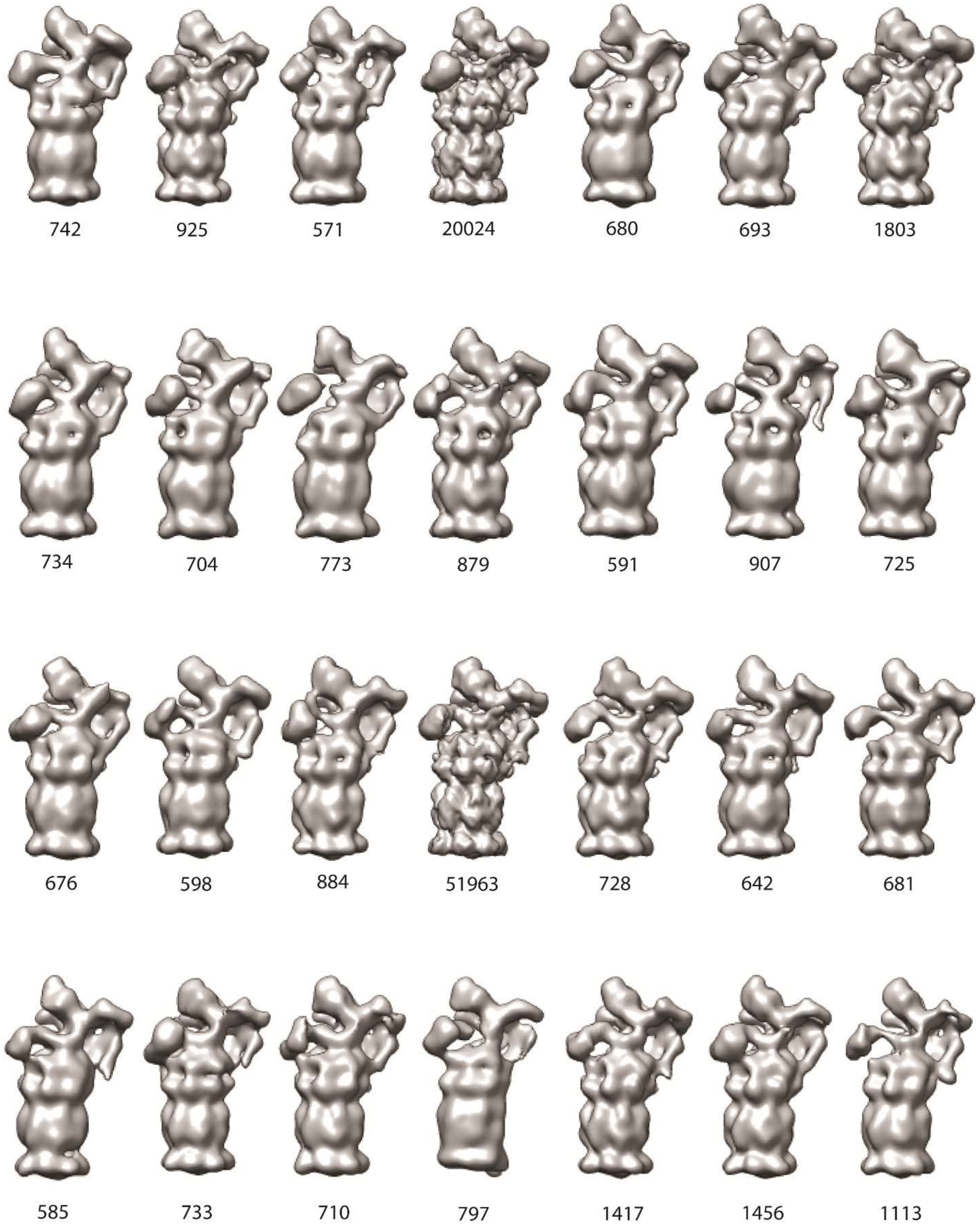
742
925
571
20024
680
693
1803
734
704
773
879
591
907
725
676
598
884
51963
728
642
681
585
733
710
797
1417
1456
1113

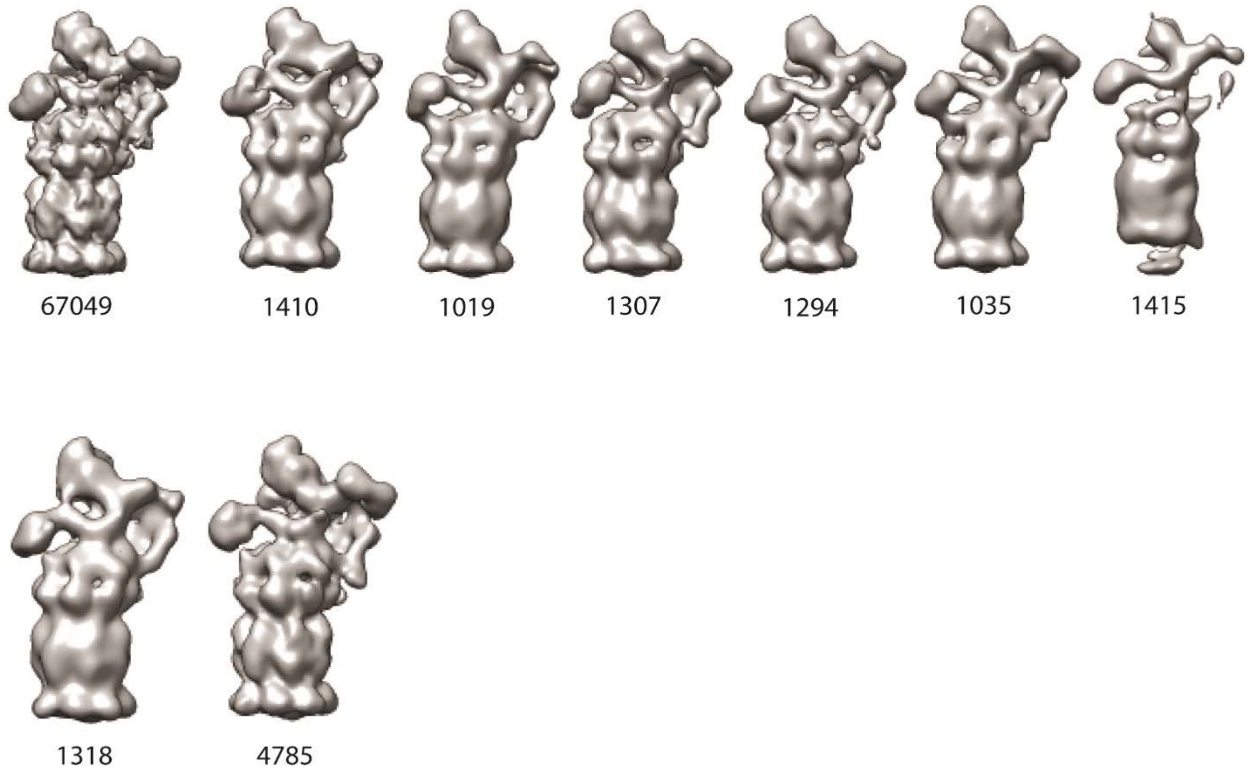

Supplementary Figure 6: Refined 3D-Classes

346 3D classes of the 26S proteasome (with and without Oprozomib) which were used to study the major modes of motion in the RP. The structures are shown with the corresponding number of particles which were used for 3D reconstruction. All 3D structures were used for the analysis of the RP mobility by PCA and to calculate the corresponding energy landscape (Supplementary Fig. 2)

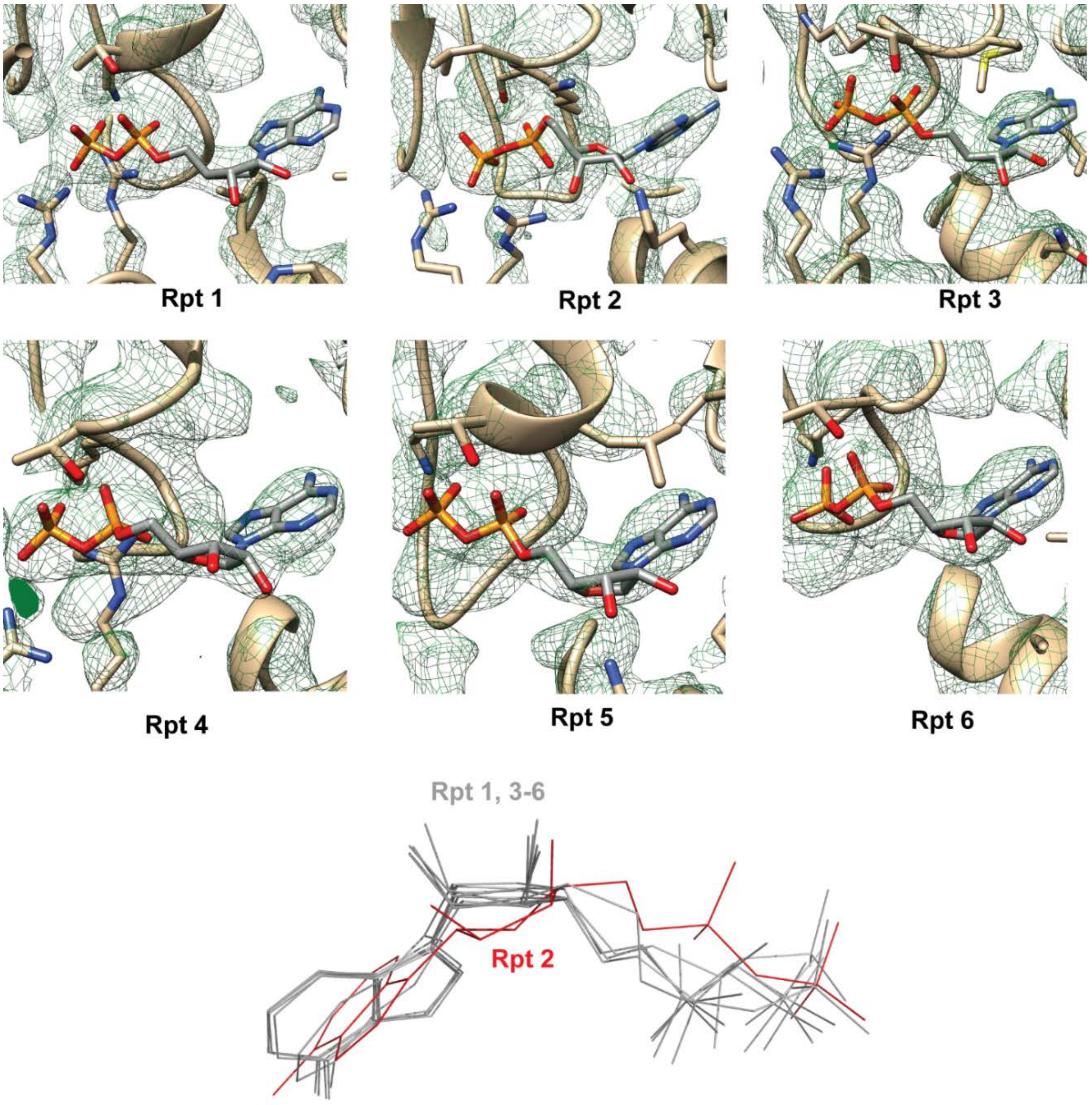

Supplementary Figure 7: Nucleotide Densities

Nucleotide densities were clearly visible for all 6 ATPase subunits. At the given resolution it cannot clearly be distinguished between ATP and ADP. Therefore, all nucleotides were built as ADP. Interestingly, the rpt2 nucleotide is the only one revealing a different sugar pucker which is in line with the strong deviation from perfect symmetry in this particular area of the ATPase.

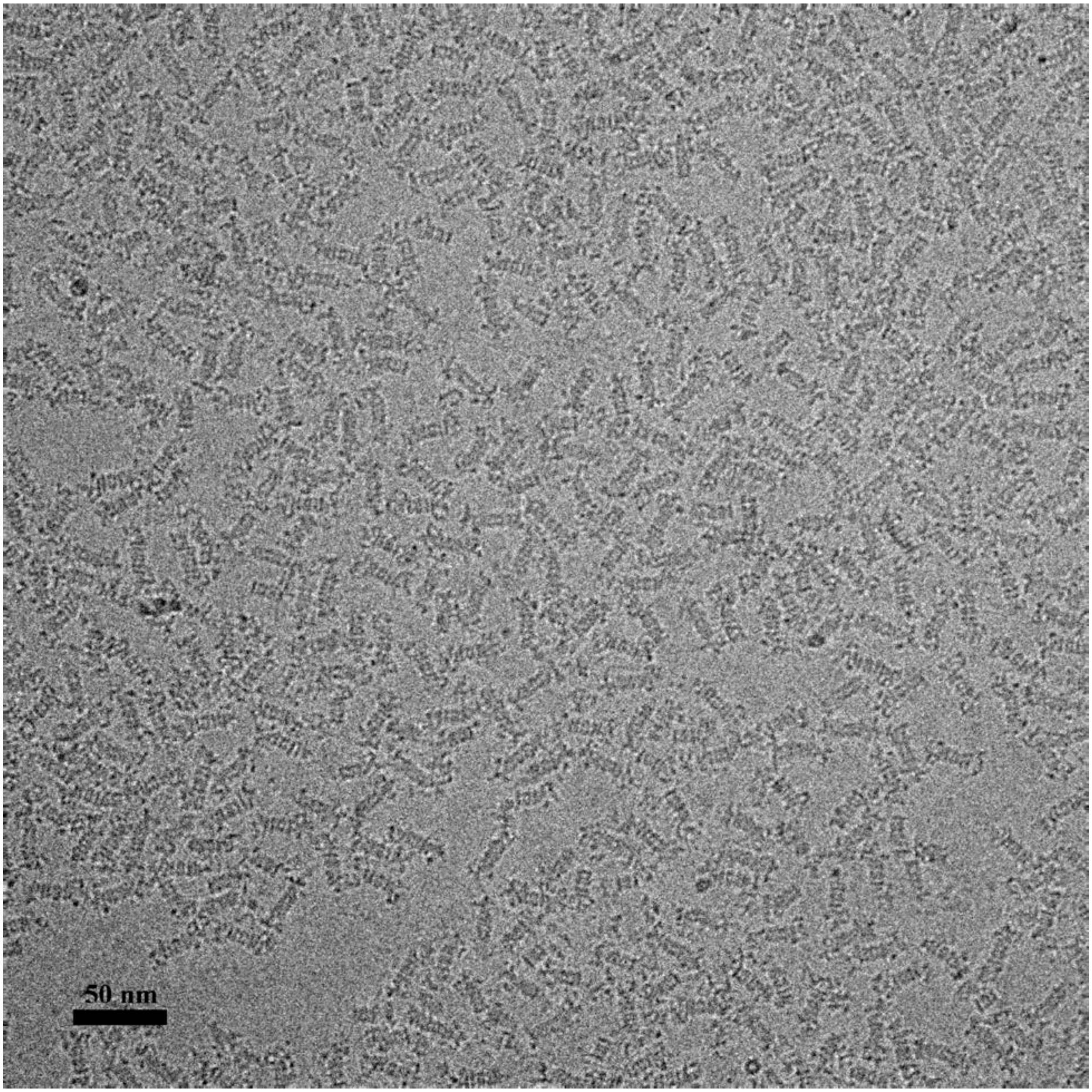

Supplementary Figure 8: Exemplary micrograph showing human 26S and 30S proteasomes

Cryo-EM micrograph of the purified proteasome. Most particles show the doubly capped 30S form. About 20 % of particles are singly capped 26S proteasomes.

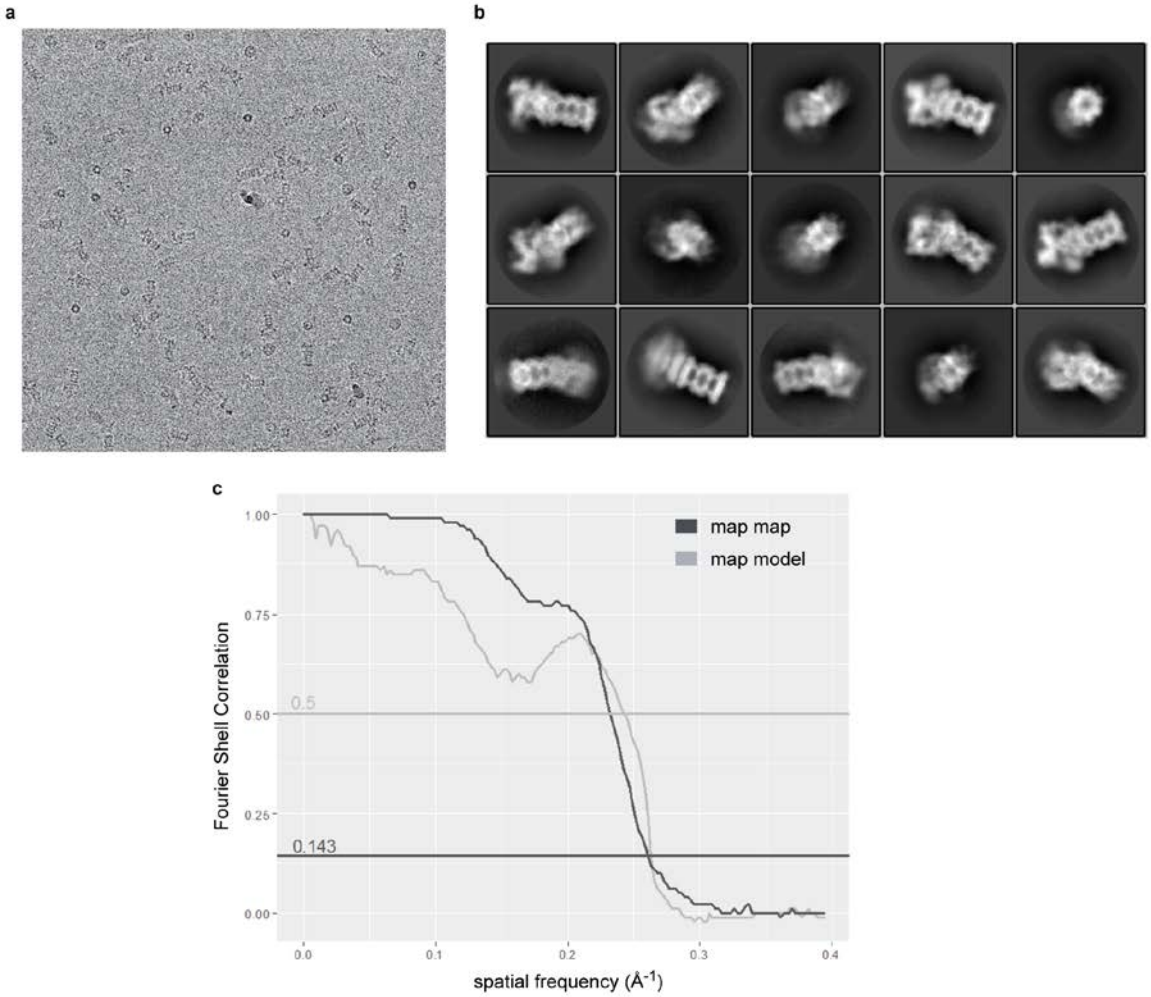

Supplementary Figure 9: Electron microscopic analysis of Oprozomib bound Proteasome

a) Micrograph depicting the various orientations of the purified 26S proteasomes.
b) Representative reference free 2D class averages.
c) Fourier Shell Correlation curves between the two half maps (map map) and the model and the map are depicted. The curve describing the correlation of the two half maps indicates the resolution of 3.8 Å using the 0.143 criterion. The curve describing the map-model correlation indicated a resolution of 4.2 Å using the 0.5 criterion.

Supplementary Table 1: 26S Proteasome nomenclature and positioning of proteins in the cryo-EM 3D structure

Yeast	Human	Chain		MW [kDa]	Activity/ Domain type	% of residues modeled		Structure Source	Reference	Note
**α1**	α1	G	U	27.40		95.12%	98.37%	5LEY	Schrader et al. (2016)	
**α2**	α2	A	O	25.80		98.71%	98.71%	5LEY	Schrader et al. (2016)	
**α3**	α3	B	P	29.50		89.66%	93.49%	5LEY	Schrader et al. (2016)	
**α4**	α4	C	Q	27.90		94.35%	96.37%	5LEY	Schrader et al. (2016)	
**α5**	α5	D	R	26.40		96.68%	96.68%	5LEY	Schrader et al. (2016)	
**α6**	α6	E	S	29.60		88.97%	91.25%	5LEY	Schrader et al. (2016)	
**α7**	α7	F	T	28.30		91.73%	94.49%	5LEY	Schrader et al. (2016)	
**β1**	β1	N	B	25.30	caspase-like protease activity	84.94%	99.02%	5LEY	Schrader et al. (2016)	
**β2**	β2	H	V	29.90	trypsin-like protease activity	79.42%	94.02%	5LEY	Schrader et al. (2016)	
**β3**	β3	I	W	22.90		100.00%	99.51%	5LEY	Schrader et al. (2016)	
**β4**	β4	J	X	22.80		97.51%	97.51%	5LEY	Schrader et al. (2016)	
**β5**	β5	K	Y	22.90	chymotrypsin-like protease activity	95.67%	98.04%	5LEY	Schrader et al. (2016)	
**β6**	β6	L	Z	26.50		88.80%	88.38%	5LEY	Schrader et al. (2016)	
**β7**	β7	M	A	29.20		81.82%	99.54%	5LEY	Schrader et al. (2016)	
**Rpt1**	S7	C		48.6	AAA+ ATPase	80.32%		Robetta (4CR2)	Unverdorben et al (2014)	

**Rpt2**	S4	D	49.2	AAA+ ATPase	75.68%	Robetta (4CR2)	Unverdorben et al (2014)	HbYX motif not resolved
**Rpt3**	S6b	E	47.3	AAA+ ATPase	89.71%	Robetta (4CR2)	Unverdorben et al (2014)	
**Rpt4**	S10b	F	44.1	AAA+ ATPase	89.46%	Robetta (4CR2)	Unverdorben et al (2014)	
**Rpt5**	S6a	G	49.1	AAA+ ATPase	82.69%	Robetta (3CF2)	Davis et al (2008)	
**Rpt6**	S8	H	45.7	AAA+ ATPase	85.71%	Robetta (4CR2)	Unverdorben et al (2014)	
**Rpn1**	S2		100.2	Scaffold	83.37%	-	-	highly flexible, not modelled
**Rpn2**	S1	I	105.9	Scaffold	35.05%	Robetta (4CR2)	Unverdorben et al (2014)	
**Rpn3**	S3	J	61	PCI	78.46%	Robetta (4D10)	Lingaraju et al	
**Rpn5**		K	52.9	PCI	92.75%	Robetta (4D10)	Lingaraju et al	
**Rpn6**	S9	L	47.4	PCI	89.07%	Robetta (4D10)	Lingaraju et al	
**Rpn7**	S10a	M	45.5	PCI	72.49%	Robetta (4D10)	Lingaraju et al	
**Rpn8**	S12	N	37.1	MPN	59.88%	Robetta (4D10)	Unverdorben et al (2014)	Highly problematic N-terminus and MPN domain
**Rpn9**	S11	O	42.9	PCI	50.80%	Robetta (4CR2)	Unverdorben et al (2014)	registration of sequence partly by secondary structure prediction
**Rpn10**	S5a	P	40.7	vWA; ubiquitin receptor	74.54%	Robetta (4F1J)	Pihlajamaa et al 2012	

**Rpn11**	S13	Q	34.6	MPN+; deubiquitination	77.74%	Robetta (4D10)	Lingaraju et al 2014	
**Rpn12**	S14	R	30	PCI	93.77%	Robetta (4CR2)	Unverdorben et al (2014)	
**Rpn13**			17.90	PRU; ubiquitin receptor		-	-	not found in structure
**sem1p**	DSS1 / SHFM1	S	8.20	intrinsically disordered	37.14%	3T5X	Ellisdon et al (2012)	

Supplementary Table 2: Model statistics

	**Human 26S proteasome + Oprozomib**
**Data collection**	
Particles	233513
Pixel Size (Å)	1.27
Defocus range (µm)	0.4-8 (mean 1.9)
Electron dose (e^-/ Å^2)	40.2
**Refinement**	
Space group	P1
a,b,c (Å)	426.27
α,β,γ (°)	90.0, 90.0, 90.0
Resolution (Å)	3.8
Wilson B (Å^2)	66.3
Map sharpening B-factor (Å^2)	-184.9
Resolution at FSC = 0.143	3.83
r_{work}	0.4526
**Rachmachandran statistics**	
Outliers	2.46 %
Favored	83.02 %
**R.m.s. deviations**	
Bond length (Å)	0.015
Bond angles (°)	1.669

**Validation**	
EMRinger score	1.94
